# Supplementary material for: Insights into fungal diversity of a shallow-water hydrothermal vent field at Kueishan Island, Taiwan by culture-based and metabarcoding analyses
Source: PLoS One. 2019 Dec 30;14(12):e0226616. doi: 10.1371/journal.pone.0226616 (PMC6936883; doi:10.1371/journal.pone.0226616)
Supplement: Attachment — Submitted filename: Culturomic and metabarcoding approaches.docx_Reviewer.docx [file pone.0226616.s003.docx]

RESEARCH ARTICLE

Insights into fungal diversity of a shallow-water hydrothermal vent field at Kueishan Island, Taiwan by culture-based and metabarcoding analysis

Ka-Lai Pang^1,^*, Sheng-Yu Guo^1^, I-An Chen^1^, Gäetan Burgaud^2^, Zhu-Hua Luo^3^, Hans U. Dahms^4^, Jiang-Shiou Hwang^1^, Yi-Li Lin^1^, Jian-Shun Huang^1^, Tsz-Wai Ho^5^, Ling-Ming Tsang^6^ , Michael Wai-Lun Chiang^7^ and Hyo-Jung Cha^1^

^1^Institute of Marine Biology and Centre of Excellence of the Oceans, National Taiwan Ocean University, Keelung, Taiwan

^2^Université de Brest, EA 3882, Laboratoire Universitaire de Biodiversité et Ecologie Microbienne, Plouzané, France

^3^Key Laboratory of Marine Biogenetic Resources, Third Institute of Oceanography, State Oceanic Administration, Xiamen, China

^4^Department of Biomedical Science and Environment Biology, College of Life Science, Kaohsiung Medical University, Kaohsiung, Taiwan

^5^School of Biological Sciences, University of Western Australia, Perth, Australia

^6^School of Biological Sciences, Chinese University of Hong Kong, Hong Kong SAR

^7^Department of Chemistry, City University of Hong Kong, Hong Kong SAR

*Corresponding author: klpang@ntou.edu.tw

Short title: Fungi of shallow hydrothermal vent at Kueishan Island (Taiwan)

Abstract

This paper reports the diversity of fungi associated with substrates collected at a shallow hydrothermal vent field at Kueishan Island, Taiwan, using both culture-based and metabarcoding methods. Culture of fungi from yellow sediment (with visible sulfur granules), black sediment (no visible sulfur granules), the vent crab *Xenograpsus testudinatus*, seawater and, animal egg samples resulted in a total of 94 isolates. Species identification based on the internal transcribed spacer regions of the rDNA revealed that the yellow sediment samples had the highest species richness with 25 taxa, followed by the black sediment (23) and the crab (13). The Ascomycota was dominant over the Basidiomycota; the dominant orders were Agaricales, Capnodiales, Eurotiales, Hypocreales, Pleosporales, Polyporales and Xylariales. *Hortaea* *werneckii* was the only common fungus isolated from the crab, seawater, yellow and black sediment samples. The metabarcoding analysis amplifying a small fragment of the rDNA (from 18S to 5.8S) recovered 7-27 species from the black sediment and 12-27 species from the yellow sediment samples of the Ascomycota and the Basidiomycota. In the yellow sediments, the dominant order was Pleosporales and this order was also dominant in the black sediment together with Sporidiobolales. Based on results from both methods, 54 and 49 species were found in the black and yellow sediments, respectively. Overall, a higher proportion of Ascomycota (~70%) over Basidiomycota was recovered in the yellow sediment and the two phyla were equally abundant in the black sediment. The top five dominant fungal orders in descending order based on species richness were Pleosporales>Eurotiales>Polyporales>Hypocreales>Capnodiales in the black sediment samples, and Polyporales>Pleosporales>Eurotiales>Capnodiales>Xylariales in the yellow sediment samples. Several pathogenic fungi of animals, including *Acremonium* spp., *Aspergillus* spp., *Fusarium* spp., *Malassezia* spp., *Hortaea werneckii*, *Parengyodontium* *album,* and *Westerdykella* *dispersa*, were recovered suggesting that these fungi may be able to cause diseases of marine animals.

**Keywords**: fungal ecology, high throughput sequencing, marine fungi, metagenomics, thermotolerant fungi.

**Introduction**

Despite numerous studies highlighting diverse marine fungi with important ecological roles such as commensals or pathogens of marine animals including corals [1] and sponges [2−3], trophic linkers between phytoplankton and zooplankton [4], or even nutrient recyclers, a relatively small percentage of described fungal species appears associated to the marine environment with 1255 species recently documented from the ocean [5]. In terms of biomass, marine fungi (together with marine protists) have been recently estimated to represent ~3% of the ~550 Gt carbon on Earth [6]. However, these estimates may be greatly underestimated due to under-sampling of diverse marine habitats. Yet, marine fungi are not represented in ocean ecosystem models [7], despite growing evidence of diverse marine fungi, especially in the deep-sea.

The deep-sea represents the largest biome on Earth, representing more than 65% of the Earth’s surface and more than 95% of the global biosphere [8]. So far, little is known about the microorganisms that live in this realm relative to our understanding of those thriving in the sunlit euphotic zone. The deep-sea encompasses a huge variety of ecological niches characterized by site-specific physical and geochemical conditions [9]. Deep-sea microorganisms, depending on the habitat, are thus facing many challenges such as elevated hydrostatic pressures, extreme temperature gradients, variable sea salt concentrations [10]. The deep subseafloor together with different deep-sea hotspots of life such as deep-sea hydrothermal vents, deep-hypersaline anoxic basins or even cold seeps have recently been reported to host active fungal communities with different ecological roles including recycling of refractory organic matter or competition with prokaryotes [11−20]. The understanding of deep-sea marine fungal diversity and distribution is mostly shaped by the methods employed. To date, the vast majority of deep-sea fungi identified mainly belongs to the Ascomycota and the Basidiomycota, independent of whether microscopy, culture or molecular tools were used. Burgaud and Edgcomb [21] have recently summarized all studies dedicated to the analysis of fungal communities from deep-sea and deep subsurface habitats and highlighted that culture-based approaches used so far have allowed the isolation of >116 species, a large majority of them being ubiquitous and affiliated to 11 fungal classes, with Eurotiomycetes, Dothideomycetes, and Microbotryomycetes being the most abundant. A “core culturable fungal diversity” consisting of 14 filamentous fungi (*Acremonium* sp., *Aspergillus glaucus* (also known as *Eurotium herbariorum*), *As. restrictus*, *As. sydowii*, *Aureobasidium pullulans*, *Cladosporium cladosporioides*, *Cl. sphaerospermum*, *Cordyceps confragosa* (also known as *Lecanicillium lecanii*), *Cyphellophora europaea*, *Exophiala dermatitidis*, *Exophiala* sp., *Penicillium chrysogenum*, *Pe. citrinum* and *Purpureocillium lilacinum*) and 3 yeasts (*Candida parapsilosis*, *Meyerozyma guilliermondii* and *Rhodotorula mucilaginosa*) was also established. Contrasted results were obtained using molecular studies. Indeed, based on 12 different studies specifically targeting deep-sea fungi, the fungal diversity was shown to be dominated by the classes Sordariomycetes, Dothideomycetes, Eurotiomycetes, Agaricomycetes, Saccharomycetes and Leotiomycetes, with *Aspergillus*, *Cryptococcus*, *Penicillium*, *Rhodotorula*, *Candida, Trichosporon, Cladosporium*, *Phoma, Exophiala, Fusarium* and *Malassezia* as the 10 most represented genera. Marine fungi affiliated to the genus *Malassezia* have only been illustrated using culture-independent methods and still represent an enigmatic lineage with unclear environmental relevance and unknown ecophysiological features. Some studies have also revealed basal fungal lineages in the deep-sea, such as the Chytridiomycota [22], and the Cryptomycota [23]. While species richness appears higher in deep-sea sediments compared to deep-sea vents, the uniqueness of these samples in terms of taxonomic composition appears higher in deep-sea vent samples, especially taking into account the recent description of new deep-sea hydrothermal vent species, for example *Candida oceani* [24] and *Yamadazyma barbieri* [25]. While deep-sea hydrothermal vents appear as unique ecological niches for fungi, no in-depth investigation of fungal communities occurring in shallow hydrothermal vents has been processed so far, to the best of our knowledge.

Kueishan Island, also known as Turtle Island, is a volcanic island lying just outside Yilan County, Taiwan (Figure 1). In the southern end of the island, there are roughly 50 hydrothermal vent systems, with depth ranging from 10 m to 80 m, constantly emitting hydrothermal fluids (fluid temperature between 48 °C and 116 °C) and volcanic gases composed of high levels of carbon dioxide and hydrogen sulfide [26−27]. Many macro-organisms have been reported near this shallow-water hydrothermal vent system including fishes, crabs, mussels, sea anemones, snails, sipunculid worms, algae and zooplankton [26, 28−29], however, knowledge on marine microorganisms including bacteria, archaea, and fungi is deficient. Recently, Wang et al. [30], using the pyrosequencing of the 16S rRNA gene, found that the Epsilonproteobacteria such as *Sulfurovum* and *Sulfurimonas* dominated the bacterial community in sediment samples collected near the hydrothermal vents at Kueishan Island. Also, they detected the presence of chemoautotrophic carbon fixation genes by the Epsilonproteobacteria in the samples suggesting their possible participation in the reductive tricarboxylic acid and the Calvin-Benson-Bassham cycles in the primary production in this extreme habitat. Complex microbial communities are supposed to occur here with either chemoautotrophic and photoautotrophic primary producers along with heterotrophs, such as fungi. Recently, Jiang et al. [31] reported the isolation of *Aspergillus* spp. from sediment samples and the hydrothermal vent crab *Xenograpsus testudinatus* at Kueishan Island, which can be seen as first hints of fungal presence in this habitat. Here, we report the first comprehensive study on the diversity of fungi on various substrates at Kueishan Island. The main objective was to investigate the diversity of fungi in sediment, seawater and organic matter collected near the hydrothermal vents using culture-based and metabarcoding approaches.

**Fig 1. Shallow hydrothermal vent field at Kueishan Island, Yilan County, Taiwan.**

Sample location: V=vent area, E=further away from vent, W=west of vent area.

**Materials and Methods**

**Collection of samples**

The location of the shallow hydrothermal vent at Kueishan Island, Yilan, Taiwan is shown in Figure 1. Samples were collected on five collection trips: 23/6/2015 (yellow (Location V, Figure 1) and black (Location W, Figure 1) sediment), 10/10/2015 (yellow sediment, seawater, the crab *Xenograpsus testudinatus* (Location V, Figure 1)), 22-23/08/2016 (yellow and black sediment, water, crabs (Location E, Figure 1)), 29-30/03/2017 (black sediment, water, crabs, animal eggs (Location V, Figure 1)) and 28/06/2017 (yellow sediment, crabs (V, Figure 1)). The sediment, seawater, crab (collected by hands of divers) and animal egg samples were immediately transferred into 50-ml universal bottles. No permit was required to collect the animal samples as the collection site is not a national park. The crab *X. testudinatus* is not an endangered/protected species in Taiwan. The samples were kept in an ice bucket and brought to the laboratory for fungal isolation and molecular analysis. Half of the sediment samples were used for isolation while the other half was freeze-dried for DNA extraction. A summary of the samples used in the isolation of fungi and the metabarcoding analysis (those with positive results) is shown in Table 1.

**Table 1. Samples used in the culture-based and metabarcoding analyses.**

Sample code: Y=yellow sediment sample, B=black sediment sample, V=vent area, E=further away from vent, W=west of vent area, 15=collected in 2015, 16=collected in 2016, 17=collected in 2017.

| **Date of sampling** | **Culture-based** | | | | | **Metabarcoding (Sample name)** | |
| --- | --- | --- | --- | --- | --- | --- | --- |
|  | **Yellow sediment** | **Black sediment** | **Crab** | **Animal egg** | **Seawater** | **Yellow sediment** | **Black sediment** |
| 23/6/2015 |  |  |  |  |  | × (Y-V-15) | × (B-W-15) |
| 10/10/2015 | × |  | × |  | × |  |  |
| 22/08/2016 |  | × |  |  |  |  | × (B-E-16) |
| 23/08/2016 | × |  |  |  |  |  |  |
| 29/03/2017 |  | × |  |  | × |  | × (B-V-17) |
| 30/03/2017 |  |  | × | × |  |  |  |
| 28/06/2017 | × |  | × |  |  | × (Y-V-17) |  |

**Isolation**

Different methods were used to isolate fungi from the seawater samples collected on different dates; undiluted/diluted seawater or concentrated (by centrifugation and resuspension in a small volume of seawater) was directly inoculated into GYPS (0.8 g/L glucose, 0.8 g/L yeast extract, 0.4 g/L peptone, 1 L natural seawater)/one-fifth strength CDS (Czapek-Dox prepared with natural seawater instead of distilled water) liquid/solid media supplemented with 0.5 g/L each of Penicillin G sodium salt and streptomycin sulfate

For the sediment samples, 1 g of sediment was directly inoculated into 50 mL GYPS and CDS liquid media supplemented with the antibiotics. The sediment samples were also serially diluted to 1×10^-3^ with 100 μL of each dilution plated on GYPS and CDS agar media supplemented with the antibiotics. For some of the sediment samples with a liquid portion (seawater), the liquid (100 μL) was directly plated on GYPS and CDS agar media with the antibiotics.

The crab samples (all dead at the time of isolation) were crushed in sterile seawater with 1 g inoculated into 50 mL GYPS and CDS liquid media with the antibiotics. The crushed crab samples were serially diluted up to 1×10^-3^ with 100 μL of each dilution plated on GYPS and CDS seawater agar media supplemented with the antibiotics. The crabs were also cut in halves with each half inoculated into GYPS and CDS liquid media with the antibiotics. The animal egg samples (collected as one clump) were directly inoculated into 50 mL GYPS and CDS liquid media with the antibiotics.

All inoculated samples were incubated at 25 °C for up to two weeks. Colonies appeared on the agar media were subcultured onto fresh GYPS agar plates as pure cultures. For the liquid media, 100 μL of the enrichments were streaked onto fresh GYPS agar plates and individual colonies growing from these plates after incubation were subcultured onto fresh GYPS agar plates as pure cultures. Visible colonies that appeared floating on top of the liquid media were also subcultured onto fresh GYPS agar plates. These pure cultures were subjected to a molecular identification based on sequencing of the internal transcribed spacer regions of rDNA including the 5.8S (ITS).

**Identification of fungal cultures**

Mycelia from 1-week old cultures were scraped from the agar plates and ground into fine powder in liquid nitrogen using a mortar and pestle. Genomic DNA was extracted using the DNeasy Plant DNA Extraction Kit (Qiagen) according to the manufacturer’s instructions. The primers ITS4 (5’−TCCTCCGCTTATTGATATGC−3’) and ITS5 (5’−GGAAGTAAAAGTCGTAACAAGG−3’), amplifying a region from 18S to 28S rDNA covering ITS1, ITS2, and 5.8S regions, were used for PCR [32]. PCR reactions were performed in 25 µL volumes containing *ca*. 20 ng DNA, 0.2 µM of each primer, 12.5 µL Gran Turismo PreMix (Ten Giga BioTech) and 1 µL of the extracted DNA. The amplification cycle consisted of an initial denaturation step of 95°C for 2 min, followed by 35 cycles of (a) denaturation (95°C for 30s), (b) annealing (54°C for 30s) and (c) elongation (72°C for 30s) and a final 10 min elongation step at 72°C. The PCR products were analyzed by agarose gel electrophoresis and sent to Genomics BioSci & Tech (New Taipei City, Taiwan) for sequencing. The sequences obtained were checked for ambiguity, assembled in MegaX [33] and submitted to the National Center for Biotechnology Information (NCBI) for a nucleotide BLAST search. The ITS sequences of the fungal isolates were deposited in NCBI with the accession numbers given in Supplementary Table 1.

**Metabarcoding study**

Total DNA was extracted from the freeze-dried sediment samples using Soil DNA Isolation Maxi Kit (Norgen Biotek) according to the manufacturer’s instructions. A nested PCR approach was used. The primers NSA3 and NLC2 [34] was used in the first round of PCR. One microliter from the first-round PCR products was used in the second round of PCR. The primers used in the second PCR were ITS1-F_KYO1 [35](Toju et al. 2012) and ITS2 [32] with adapter sequences added on the 5’ end of the primers.

PCR reactions were performed in 25 µL volumes containing *ca*. 20 ng DNA, 0.2 µM of each primer, 12.5 µL Gran Turismo PreMix (Ten Giga BioTech) and 1 µL first-round PCR product. The amplification cycle consisted of an initial denaturation step of 95°C for 2 min followed by 35 cycles of (a) denaturation (95°C for 30s), (b) annealing (54°C for 30s) and (c) elongation (72°C for 30s) and a final 10 min elongation step at 72°C. Five PCR reactions were performed for each sample and pooled. The pooled sample was run on a 1% agarose gel, gel-purified using EasyPure PCR/Gel Extraction Kit (Bioman Scientific Co., Ltd.) and sent to Genomics BioSci & Tech (New Taipei City, Taiwan) for Illumina MiSeq sequencing. Negative PCR controls (water control) were run to detect the contamination of the PCR ingredients.

Most of the following bioinformatics processes were run in QIIME 1.9.0 [36]. The raw sequences were filtered with a phred score ≥Q29 (a base call accuracy of ≥99.87%). The raw reads were paired into single reads and adaptors, primers and barcode sequences were removed using QIIME with the script split_library.py [36]. Clustering was performed using uclust v1.2.22q [37] in QIIME [36]. The reads were processed with UCHIME [38] to remove chimeric sequences. Assigning Operation Taxonomic Units (OTUs) and taxonomic assignments were performed with an open-reference OTU picking approach against the UNITE database in QIIME [36]. A similarity threshold of 97% was adopted. Taxonomic assignment of representative OTUs was run at a 0.97 confidence threshold against the UNITE ITS1 database with UNITE 7.2 reference OTU database ("UNITE+INSD" dataset) using the assignTaxonomy method [39].

**Statistical analysis**

Total number of isolates (total abundance, N), richness (total number of taxa in the community, S), species richness (Margalef), Shannon-Wiener diversity index, Pielou's evenness and Simpson diversity indices (Simpson's Index, Simpson's Index of Diversity, Simpson's Reciprocal Index) were calculated by building the respective equations in Microsoft Excel.

Rarefaction and extrapolation sampling curves were computed and plotted to estimate sample completeness (sample coverage) in R package iNEXT (iNterpolation/EXTrapolation) with the 95% lower and upper confidence limits for the isolation and metabarcoding data [40]. Principle component analysis (PCA) was used to analyze trends in fungal species composition of the sediments (black or yellow) collected from the three collection spots (vent region, east of Turtle Island, west of vent area, Figure 1) between 2015 and 2017 and calculated by the R software using the R function prcomp() [41].

**Results**

**Culturable diversity of fungi**

A total of 94 isolates was cultured from the crab *Xenograpsus* *testudinatus*, sediment, seawater and animal egg samples (Supplementary Table 1). These fungi were identified based on comparisons of their ITS sequences with those in the GenBank database. The identity of the fungi was referred in most cases to a species name and in some cases to a genus, based on the top BLAST results with the highest score, in terms of high query coverage (%) and identity (%). When the top BLAST results were unidentified/uncultured fungi, the next result with a name was used if the % query coverage and % identity were high (≥95%); if these figures were low, the identity was only referred to the family, order, class or phylum.

Table 2 and Figure 2 summarize the fungal species richness obtained from the different samples. Sediment samples had the highest species richness: 25 taxa from the yellow sediment and 23 taxa from the black sediment. Thirteen taxa were isolated from the crab samples. Only two species each were isolated from the seawater and the animal egg samples. A higher proportion of the Ascomycota was isolated from the black sediment samples than the yellow sediment samples based on the total species richness (>70%, Figure 3a, d). At the class level, 5 different classes of fungi were isolated from the black and 6 from the yellow sediment samples (Figure 3b, e). At the order level, the fungi isolated from the yellow sediment could be referred to 11 different fungal orders, followed by black sediment (10), crabs (7), seawater (2) and animal egg (1) samples (Table 2, Figure 3c, f). Dominant orders based on species richness were Agaricales, Capnodiales, Eurotiales, Hypocreales, Pleosporales, Polyporales and Xylariales. Species of the Hypocreales were isolated from all types of samples (Table 2).

**Fig 2. Venn diagram.**

The number represents fungal species richness in yellow sediment, black sediment, vent crab *Xenograpsus* *testudinatus*, animal egg and seawater samples and between different samples.

**Table 2. List of fungal species from the culturomics.**

Fungal taxa isolated from crab, black sediment, yellow sediment, seawater and animal egg samples collected near/at the hydrothermal vents at Kueishan Island, Taiwan.

| **Crab** | **Black sediment** | **Yellow sediment** | **Seawater** | **Animal eggs** |
| --- | --- | --- | --- | --- |
| **Ascomycota** | | | | |
| Capnodiales | Capnodiales | Capnodiales | Capnodiales | Hypocreales |
| *Hortaea werneckii** | *Hortaea werneckii** | *Fodinomyces uranophilus* | *Hortaea werneckii** | *Cordyceps takaomontana* |
| Eurotiales | Eurotiales | *Hortaea werneckii** | Hypocreales | *Fusarium* sp. |
| *Aspergillus sydowii* | *Aspergillus* sp. 2 | Eurotiales | *Parengyodontium album** |  |
| *Aspergillus terreus** | *Aspergillus taichungensis* | *Aspergillus aculeatus* |  |  |
| *Aspergillus ungillus* | *Aspergillus terreus** | *Aspergillus terreus** |  |  |
| *Penicillium citreosulfuratum* | *Penicillium citrinum* | *Penicillium oxalicum* |  |  |
| Hypocreales | Glomerellales | *Penicillium* sp. |  |  |
| Hypocreales sp. | *Gibellulopsis nigrescens* | *Penicillium sumatrense* |  |  |
| Microascales | Hypocreales | Hypocreales |  |  |
| *Microascus brevicaulis* | *Parengyodontium album** | *Acremonium brunnescens* |  |  |
| Saccharomycetales | *Trichoderma harzianum** | *Acremonium citrinum* |  |  |
| *Candida oceani* | Microascales | *Acremonium felinum* |  |  |
| Xylariales | Microascales sp. | *Trichoderma harzianum** |  |  |
| *Hypoxylon monticulosum* | Pleosporales | Ophiostomatales |  |  |
| *Peroneutypa scoparia** | *Allophoma tropica* | *Sporothrix* sp. |  |  |
| *Xylaria* sp. | *Didymella* sp./*Phoma* sp. | Pleosporales |  |  |
| *Incertae sedis* | *Leptosphaeria* sp. | *Curvularia clavata* |  |  |
| *Tritirachium* sp. | *Microsphaeropsis arundinis* | *Westerdykella dispersa** |  |  |
|  | Pleosporales sp. | Saccharomycetales |  |  |
|  | *Westerdykella dispersa** | *Meyerozyma guilliermondii* |  |  |
|  | Sordariales | Xylariales |  |  |
|  | *Chaetomium globosum* | *Peroneutypa scoparia** |  |  |
|  | Xylariales | *Xylaria apiculata* |  |  |
|  | *Arthrinium arundinis* | *Xylaria curta* |  |  |
|  | *Arthrinium hydei* | *Incertae sedis* |  |  |
|  | *Incertae sedis* | Ascomycetes sp. |  |  |
|  | Pezizomycotina sp. |  |  |  |
| **Basidiomycota** | | | | |
| Agaricales | Agaricales | Agaricales |  |  |
| *Chondrostereum* sp.* | *Chondrostereum* sp.* | *Chondrostereum* sp.* |  |  |
|  | Polyporales | Cystobasidiales |  |  |
|  | *Bjerkandera adusta* | *Cystobasidium minutum* |  |  |
|  | *Cerrena* sp.* | Hymenochaetales |  |  |
|  |  | *Tropicoporus* sp. |  |  |
|  |  | Polyporales |  |  |
|  |  | *Cerrena* sp.* |  |  |
|  |  | *Earliella scabrosa* |  |  |
|  |  | *Porostereum* sp. |  |  |

**Fig 3. Diversity of fungi (based on percentage of species richness) in the black and yellow sediment samples.**

Classification at phylum, class and order levels using culture-based (Culture) and metabarcoding (HTS) approaches. Total combines the results from both methods.

Concerning the common fungal species between the different sample types, *Hortaea* *werneckii* was the only fungus isolated from four types of samples, i.e. crab, seawater, yellow and black sediment samples (Table 2). Two species were common between the crab, yellow and black sediment samples (*Aspergillus terreus*, *Chondrostereum* sp.). One common species were isolated from the crabs and the yellow sediment (*Peroneutypa* *scoparia*) and the black sediment and seawater samples (*Parengyodontium* *album*). Three other species were isolated from both the black and yellow sediments (*Cerrena* sp., *Trichoderma* *harzianum*, *Westerdykella* *dispersa*).

**Metabarcoding analysis of sediments**

Twenty-two sediment subsamples from the five collection trips were subjected to the DNA extraction and the PCR. Positive PCR products were obtained from only 13 sediment subsamples (4 yellow and 9 black sediment subsamples) collected on 23/06/2015, 22-23/08/2016, 29/03/2017 and 28/06/2017 and subsequently sent for the high throughput sequencing. The number of sequences in the 13 subsamples ranged from 15,211 to 113,956, with a total of 773,112 sequences and an average of 59,470 sequences per sample (results not shown). The PCA analysis based on operational taxonomic unit (OTU) and read number shows that the first axis (PC1) described 40.5% of the variation in the species composition between sediment samples while the second axis (PC2) described 37.07% of the variation, giving a total of 77.57% of the variation in the dataset (Figure 4). From the score plot, sediment samples with a similar species composition clustered together. Generally, there was a gradient of species composition along PC1 axis, decreased from the black sediment samples collected at site E (further away from vent) to site W (west of vent area), and to site V (vent area); the lowest was the yellow sediment samples collected at site V (vent area). Overall, yellow sediment samples of the vent area had the lowest species composition in both PCA1 and PCA2 when compared with other sediment samples (black sediments, regardless of sites).

**Fig 4. Principle component analysis (PCA) of the 13 sediment subsamples analyzed with metabarcoding analysis.**

Sample code: V=vent area, E=further away from vent, W=west of vent area; B=black sediment sample, Y=yellow sediment sample; 15=collected in 2015, 16=collected in 2016, 17=collected in 2017; a-e=subsample number.

Reads from the 13 subsamples were combined and referred to 5 samples based on the date of collection and sample type. The rarefaction analysis shown in Figure 5 reveals that species diversity had reached saturation for all 5 samples. The cluster analysis (Bray-Curtis similarity) based on the richness and abundance (read number) of the fungi in the five samples is shown in Figure 6. The samples of the same type (black, yellow sediment) and collected on the same dates did not group.

**Fig 5. Rarefaction analysis of the five sediment samples based on sequence reads.**

Sample code: B=black sediment sample, Y=yellow sediment sample; V=vent area, E=further away from vent, W=west of vent area; 15=collected in 2015, 16=collected in 2016, 17=collected in 2017.

**Fig 6. Bray-Curtis similarity of the five sediment samples based on sequence reads.**

Sample code: B=black sediment sample, Y=yellow sediment sample; V=vent area, E=further away from vent, W=west of vent area; 15=collected in 2015, 16=collected in 2016, 17=collected in 2017.

Excluding the sequences belonging to the Fungi/Unassigned category, species richness of fungi in the black sediment samples was in the range of 7-27 OTUs, in the yellow sediment samples in the range of 12-27 OTUs (species/taxa hereafter) (results not shown). In the black sediment samples, the Ascomycota and the Basidiomycota were equally abundant but the Ascomycota was dominant over the Basidiomycota in the yellow sediment samples (Figure 7a, d). At the class and order levels, the fungi were more diverse in the black sediments than the yellow sediments; 10 classes and 17 orders in the black sediments (Figure 7b, e) while only 7 classes and 12 orders in the yellow sediments (Figure 7c, f). In the yellow sediments, the dominant classes were the Dothideomycetes (56.18% in abundance) and Agaricomycetes (9.49%) while the other classes constituted only small fractions of the sequences (~1%) (Figure 7e). The dominant orders in the yellow sediments were Pleosporales (55.62%) and Polyporales (9.19%) and the rest took up only small percentages (Figure 7f). In the black sediments, the Dothideomycetes (13.09%), the Microbotryomycetes (10.90%) and the Agaricomycetes (7.19%) were the dominant classes (Figure 7b) and the Sporidiobolales (10.90%), the Pleosporales (10.31%) and the Chaetothyriales (4.11%) were the dominant orders (Figure 7c).

**Fig 7. Diversity of fungi (based on percentage of species richness) in the black (a-c) and yellow (d-f) sediment samples.**

Classification at phylum, class, and order levels using metabarcoding approach.

**Comparison of culture and HTS in sediments**

Figure 3 shows the diversity of fungi isolated from the black and yellow sediment samples at the phylum, class and order levels from the isolation and metabarcoding methods (excluding unidentified/unknown fungi) based on species richness. Generally, the Ascomycota was dominant over the Basidiomycota in the isolation approach (Figure 3a, d). More diverse classes and orders, on the other hand, were obtained from the metabarcoding method (Figure 3b-c, e-f). A comparison of the common taxa in the black and yellow sediments at the class, order and genus levels between the two methods is shown in Figure 8. A higher richness was found in the black sediment samples and in the metabarcoding analysis at the class, order and genus levels.

**Fig 8. Fungal richness.**

Comparison of fungal richness at class, order and genus levels in black and yellow sediment samples using culturomics and metabarcoding analysis.

**Total diversity of fungi in sediments**

Overall, the proportion of Ascomycota (~70%) to Basidiomycota in the black and yellow sediment samples was similar (Figure 3a, d). At the class level, the fungi in the black sediment samples can be referred to 10 different fungal classes and the dominant classes were Agaricomycetes (Basidiomycota), Dothideomycetes and Sordariomycetes (Ascomycota) (Figure 3b). These three classes among six other classes were also dominant in the yellow sediment samples (Figure 3e). Nineteen different fungal orders were discovered from the black sediment samples (Figure 3c) while ten from the yellow sediment samples (Figure 3f). The top five dominant fungal orders in descending order based on species richness were Pleosporales>Eurotiales>Polyporales>Hypocreales>Capnodiales in the black sediment samples, and Polyporales>Pleosporales>Eurotiales>Capnodiales>Xylariales in the yellow sediment samples.

Table 3 lists the species of fungi based on the results of the culture-based and the metabarcoding analyses of the black and yellow sediment samples, excluding those taxa only classified above the genus level. A total of 54 and 49 species were found in the black and yellow sediments, respectively, excluding the various unknown *Aspergillus* spp. in the black sediment. Twenty-eight species were common between the two sediment types. Species of *Colletotrichum*, *Fusarium* and *Malassezia* were only identified in the black sediment samples, while species of *Acremonium* and *Xylaria* only in the yellow sediment samples.

**Table 3. List of fungal species in the sediment samples.**

The list was constructed based on taxa from both culture-based and metabarcoding methods. Common taxa in both sediment types are marked with an asterisk.

| **Black sediment** | **Yellow sediment** |
| --- | --- |
| **Ascomycota** | |
| Capnodiales | Capnodiales |
| *Cladosporium delicatulum** | *Cladosporium delicatulum** |
| *Cladosporium sphaerospermum** | *Cladosporium sphaerospermum** |
| *Cladosporium* sp.* | *Cladosporium* sp.* |
| *Hortaea werneckii** | *Hortaea werneckii** |
| Chaetothyriales | *Fodinomyces uranophilus* |
| *Exophiala xenobiotica** | Chaetothyriales |
| Dothideales | *Capronia semi-immersa* |
| *Aureobasidium* sp.* | *Exophiala xenobiotica** |
| Eurotiales | Dothideales |
| *Aspergillus penicillioides** | *Aureobasidium* sp.* |
| *Aspergillus taichungensis* | Eurotiales |
| *Aspergillus terreus** | *Aspergillus aculeatus* |
| *Aspergillus* spp. | *Aspergillus penicillioides** |
| *Penicillium citrinum* | *Aspergillus terreus** |
| Glomerellales | *Penicillium oxalicum* |
| *Colletotrichum brasiliense* | *Penicillium sumatrense* |
| *Colletotrichum gloeosporioides* | *Penicillium* sp. |
| *Gibellulopsis nigrescens* | Hypocreales |
| Hypocreales | *Acremonium brunnescens* |
| *Fusarium solani* | *Acremonium citrinum* |
| *Fusarium* sp. | *Acremonium felinum* |
| *Parengyodontium album* | *Acremonium polychromum* |
| *Simplicillium obclavatum* | *Trichoderma harzianum** |
| *Trichoderma harzianum** | *Trichoderma lixii** |
| *Trichoderma lixii** | Ophiostomatales |
| *Trichoderma* sp. | *Sporothrix* sp. |
| Pleosporales | Pleosporales |
| *Allophoma tropica* | *Curvularia clavata* |
| *Alternaria* sp. | *Preussia persica** |
| *Didymella* sp./*Phoma* sp. | *Pyrenochaetopsis leptospora** |
| *Leptosphaeria* sp. | *Pyrenochaetopsis* sp. |
| *Microsphaeropsis arundinis* | *Sclerostagonospora phragmiticola** |
| *Preussia persica** | *Westerdykella dispersa** |
| *Pyrenochaetopsis leptospora** | Saccharomycetales |
| *Roussoella solani* | *Meyerozyma guilliermondii* |
| *Sclerostagonospora ericae* | Xylariales |
| *Sclerostagonospora phragmiticola** | *Arthrinium* sp.* |
| *Stagonospora neglecta* | *Hypoxylon monticulosum** |
| *Westerdykella dispersa** | *Nigrospora oryzae** |
| Sordariales | *Peroneutypa scoparia* |
| *Chaetomium globosum* | *Xylaria apiculata* |
| Xylariales | *Xylaria curta* |
| *Arthrinium arundinis* |  |
| *Arthrinium hydei* |  |
| *Arthrinium* sp.* |  |
| *Hypoxylon monticulosum** |  |
| *Nigrospora oryzae** |  |
| **Basidiomycota** | |
| Agaricales | Agaricales |
| *Chondrostereum* sp.* | *Chondrostereum* sp.* |
| *Coprinopsis urticicola* | *Psilocybe coprophila** |
| *Psilocybe coprophila** | *Psilocybe* sp.* |
| *Psilocybe inquilina* | Cystobasidiales |
| *Psilocybe* sp.* | *Cystobasidium minutum* |
| Geminibasidiales | Geminibasidiales |
| *Geminibasidium* sp.* | *Geminibasidium* sp.* |
| Malasseziales | Hymenochaetales |
| *Malassezia globosa* | *Tropicoporus* sp. |
| *Malassezia restricta* | Polyporales |
| Polyporales | *Bbjerkandera adusta** |
| *Bjerkandera adusta** | *Cerrena* sp.* |
| *Cerrena* sp.* | *Earliella scabrosa* |
| *Phanerochaete tuberculata* | *Phlebia chrysocreas** |
| *Phlebia chrysocreas** | *Porostereum* sp. |
| *Rigidoporus* sp.* | *Rigidoporus* sp.* |
| *Trametes cubensis** | *Trametes cubensis** |
| Russulales | Russulales |
| *Peniophora* sp.* | *Peniophora* sp.* |
| Sporidiobolales | Sporidiobolales |
| *Rhodotorula mucilaginosa** | *Rhodotorula mucilaginosa** |

**Discussion**

**Environmental relevance of fungal isolates**

The vent crab *Xenograpsus testudinatus*, sediment (yellow and black), seawater and animal eggs were collected at/near the hydrothermal vent system of the Kueishan Island, Taiwan for the isolation of fungi. The sediment samples yielded the highest fungal richness, supporting recent studies revealing complex fungal communities associated with different marine sediments, either shallow [42−43] or deep [19−20]. Marine sediments seem to represent a reservoir of fungi, most of them being ubiquitous [44]. These results may also be linked to the fact that more sediment samples were collected than the other samples, i.e. crabs, seawater and animal eggs and consequently, more species were isolated from the sediment samples.

Thirteen species in seven orders of fungi were isolated from the crab *Xenograpsus testudinatus* with *Aspergillus* as the most retrieved genus on the crab. The association between such fungi and the crab is unclear and an in-depth study is required to get insights into real or erratic association, e.g. by characterizing biofilm on carapace of the crab, by examining more crab samples at the same site to see whether fungi can lead to diseases (necrosis for example) or by examining the ability of fungi isolated on dead crabs to synthesize specific hydrolytic enzymes such as chitinases.

*Hortaea werneckii* is likely to be a common fungus at/near the hydrothermal vent system at Kueishan Island as it was the only fungus cultured from the sediment, seawater and crab samples. *Hortaea werneckii* is possibly a marine fungus, although it was not listed as a marine species [45]. This species is halophilic [46] and has previously been reported from a hydrothermal vent at the Atlantic Ocean [14] and scuba diving equipment [47]. *Hortaea* *werneckii* was reported to cause tinea nigra in humans [48] and may occur as an opportunistic pathogenic fungus but whether this fungus can cause diseases of vent animals requires a thorough investigation.

Seven common orders (Agaricales, Capnodiales, Eurotiales, Hypocreales, Pleosporales, Polyporales, and Xylariales) of fungi were isolated from the yellow and black sediments and six species (*Aspergillus* *terreus*, *Cerrena* sp., *Chondrostereum* sp., *Hortaea* *werneckii*, *Trichoderma* *harzianum*, *Westerdykella* *dispersa*) were cultured from both sample types. Fungi of the Agaricales, Capnodiales, Eurotiales, Hypocreales, and Polyporales are common in the marine environment, especially seawater and sediment [45]. The Xylariales has been isolated as endophytes of marine-adapted mangrove plants, seagrasses and macroalgae [49]. However, *Peroneutypa scoparia* of the Xylariales was reported to be an endophyte of plants and also a wood inhabitant [50] but it is unknown in the marine environment. No yeasts were isolated from the black sediment and only two marine yeast species, *Cystobasidium* *minutum* and *Meyerozyma* *guilliermondii*, were isolated from the yellow sediment. Yeasts are common in the marine environment, especially basidiomycetous yeasts [51] and the low number of yeasts isolated may be related to the isolation methods. Yeasts were covered by dense mycelia of filamentous fungi on the isolation plates which impeded their isolation. Sequences of the yeasts *Geminibasidium* sp. and *Rhodotorula* *mucilaginosa* recovered through the metabarcoding analysis confirm the presence of other yeast species in both sediment types.

**Molecular diversity of fungi**

Ascomycota and Basidiomycota were the only phyla identified from sequences of the metabarcoding analysis. No basal fungal lineages, such as the Chytridiomycota and the Cryptomycota, were obtained, although such lineages are common in the marine environment [52]. This might be due to the primer bias towards the amplification of the Ascomycota and the Basidiomycota in the PCR reactions.

The fungal community of the five sediment samples analyzed through the metabarcoding analysis did not form separate clusters based on the date of collection and sample types, suggesting that no temporal variation or substrate-specificity seem to occur. However, the whole picture (Figure 3) strongly highlights that each type of sediment harbor specific fungal communities.

Over half of the total reads in the yellow sediment belonged to the Pleosporales (55.62%) of the Dothideomycetes (56.18%) due to the high number of reads belonging to *Westerdykella dispersa* (55.11%). The role of *W. dispersa* near the hydrothermal vent is unknown but this species was previously reported from the marine sediment of the South China Sea [53]. The proportion of the different dominant classes (Agaricomycetes, Microbotryomycetes, Dothideomycetes, ~10%) and orders (Sporidiobolales, Pleosporales, ~10%) in the black sediment was comparable.

On the sediment samples in which both the isolation and metabarcoding methods were applied, no common species of fungi were recovered from the black sediment samples collected on 22/08/2016 and 29/03/2017 and the yellow sediment samples collected on 28/06/2017. Culture-dependent methods favor fast-growing fungi such as *Asperigllus* spp. and *Penicillium* spp. (Table 2), species that may not be dominant in the samples. The diverse classes and orders of fungi recovered from the metabarcoding method confirm the pitfalls of using isolation as the sole method to studying the diversity of fungi.

**Putative ecological roles**

Based on the results from the metabarcoding analysis and the culture-based approach, the number of fungal species from the yellow (49) and black (54) sediments was comparable. Among these fungi, the majority has been classified as terrestrial taxa while a few can be recognized as marine [45] (Table 3). Species richness of the Ascomycota was higher than the Basidiomycota, supporting the results of other studies highlighting the dominance of ascomycetes in the marine environment. The dominant classes (i.e. Agaricomycetes, Dothideomycetes and Sordariomycetes) and orders (i.e. Capnodiales, Eurotiales, Hypocreales, Pleosporales, Polyporales, Xylariales) in both the yellow and black sediment types were similar. Most marine Pleosporales are intertidal mangrove species [45, 54−55]. As discussed above, basidiomycetous yeasts are common in the marine environment but not many filamentous basidiomycetes are known in the marine environment [45] and it may be related to salinity sensitivity [56]. The high occurrence of the filamentous basidiomycetes in this study may suggest that spores of the terrestrial Polyporales (*Bjerkandera adusta*, *Cerrena* sp., *Phanerochaete tuberculate*, *Phlebia chrysocreas*, *Rigidoporus* sp. and *Trametes cubensis*) and Agaricales (*Chondrostereum* sp.) deposit in the marine sediment. Four species are described in *Chondrostereum* and *C. purpureum* can cause the silverleaf disease, which causes the death of plants and its spores are dispersed by air [57]. Fröhlich-Nowoisky et al. [58] examined the diversity of fungi in continental, coastal and marine air; the Basidiomycota constituted 41% and 28% of the total fungal propagules in coastal and marine air, respectively and the dominant class was Agaricomycetes in both air types. It is likely that spores of the Agaricomycetes settle into the ocean and eventually sink to the seabed but they play no role in the sediment. The shallow nature of the Kueishan Island vent field and its close proximity to land strongly support the idea of terrestrial spore/hyphae dissemination from land to the marine environment. Although speculative, such preliminary results pave the road for a complementary fungal-focused metatranscriptomic approach to delve deeper into marine fungal functions at the mRNA expression level. In the same study [58], the Ascomycota belonging to the Dothideomycetes, Eurotiomycetes, Leotiomycetes and Sordariomycetes was dominant in the coastal and marine air and it may also explain the dominance of the Dothideomycetes, Eurotiomycetes, and Sordariomycetes in the sediment.

Species of *Colletotrichum*, *Fusarium* and *Malassezia* were only identified in the black sediment samples, the same is true for species of *Acremonium* and *Xylaria* in the yellow sediment samples. *Colletotrichum* is a genus of common plant disease fungi including a range of agricultural plants and of endophytes of terrestrial plants [59]. Currently available evidence does not suggest *Colletotrichum* play a role in the marine sediment, same for *Xylaria* spp. . Members of the genus *Fusarium* are also well known in terrestrial habitats as common soil fungi or plant pathogens. However, some *Fusarium* species have true roles in the marine environment; for example *F. oxysporum* has been reported to associate to marine mammals like dolphins and whales [60], causing dermatitis and systemic lesions sometimes leading to mortalities, and also known as a denitrifying species in oxygen-depleted regions in the marine environment [61]. The genus *Malassezia* is ecologically hyper-diverse and among the most widespread fungi in the marine environment. DNA signatures of *Malassezia* and *Malassezia*-like sequences have been retrieved from numerous contrasted marine habitats, including sponges and deep-sea sediments [10]. Complementary rRNA biosignatures have revealed metabolic activities of this genus in deep-sea sediments [15, 18]. The detection of DNA signatures of *Malassezia* in the black sediment at the hydrothermal vents of Kueishan Island is another proof of *Malassezia* occurrence in the marine environment and definitely support the idea that *Malassezia* members represent a relevant target taxon to isolate into culture in the coming years to better assess their ecophysiological features.

This study, as in a number of other studies on fungal diversity of marine sediment, recovered a number of fungal taxa with potential animal pathogenicity, including *Acremonium* spp., *Aspergillus* spp., *Fusarium* spp., *Malassezia* spp., *Hortaea werneckii*, *Parengyodontium* *album,* and *Westerdykella* *dispersa*. *Hortaea werneckii*, *P. album* and *W. dispersa* [62−64] and whether these species can cause diseases of the vent animals (i.e. the vent crab *Xenograpsus testudinatus*) is unknown at Kueishan Island. More research is required to determine whether any of the fungi recovered from the marine sediment can cause diseases of bottom-dwelling animals and impact this specific food web.

**Supporting Information**

**S1 File.** BLAST search results of the fungi isolated from sediment, seawater, the vent crab *Xenograpsus testudinatus* and animal egg samples collected at the shallow hydrothermal vent field, Kueishan Island, Taiwan.

**References**

1. Alker AP, Smith GW, Kim K. Characterization of *Aspergillus sydowii* (Thom *et* Church), a fungal pathogen of Caribbean sea fan corals. Hydrobiologia 2001;460: 105-111.
2. Gao Z, Li B, Zheng C, Wang G. Molecular detection of fungal communities in the Hawaiian marine sponges *Suberites* *zeteki* and *Mycale* *armata*. Appl Environ Microbiol. 2008;74: 6091-6101.
3. Ein-Gil N, Ilan M, Carmeli S, Smith GW, Pawlik JR, Yarden O. Presence of *Aspergillus* *sydowii*, a pathogen of gorgonian sea fans in the marine sponge *Spongia* *obscura*. ISME J. 2009;3: 752-755.
4. Kagami M, Miki T, Takimoto G. Mycoloop: chytrids in aquatic food webs. Front Microbiol. 2014;5: 166.
5. Jones EBG, Pang KL, Abdel-Wahab M, Scholz B, Hyde KD, Boekhout T, et al. An online resource for marine fungi. Fungal Divers. 2019;DOI: 10.1007/s13225-019-00426-5
6. Bar-On YM, Phillips R, Milo R. The biomass distribution on Earth. PNAS 2018;115: 6506-6511.
7. Worden AZ, Follows MJ, Giovannoni SJ, Wilken S, Zimmerman AE, Keeling PJ. Environmental science. Rethinking the marine carbon cycle: factoring in the multifarious lifestyles of microbes. Science 2015;347: 1257594.
8. Herring P. The biology of the deep ocean. Oxford: Oxford University Press, England; 2001.
9. Gaboyer F, Burgaud B, Edgcomb V. The deep subseafloor and biosignatures. In: Cavalazzi B, Westall F, editors. Biosignatures for Astrobiology. Advances in Astrobiology and Biogeophysics. Cham: Springer; 2019. pp. 87-109.
10. Rédou V, Kumar A, Hainaut M, Henrissat B, Record E, Barbier G, et al. Draft genome sequence of the deep-sea ascomycetous filamentous fungus *Cadophora malorum* Mo12 from the Mid-Atlantic Ridge reveals its biotechnological potential. Genome Announc. 2016;4: e00467-16.
11. Takishita K, Tsuchiya M, Reimer JD, Maruyama T. Molecular evidence demonstrating the basidiomycetous fungus *Cryptococcus curvatus* is the dominant microbial eukaryote in sediment at the Kuroshima Knoll methane seep. Extremophiles 2006;10: 165-169.
12. Takishita K, Yubuki N, Kakizoe N, Inagaki Y, Maruyama T. Diversity of microbial eukaryotes in sediment at a deep-sea methane cold seep: surveys of ribosomal DNA libraries from raw sediment samples and two enrichment cultures. Extremophiles 2007;11: 563-576.
13. Burgaud G, Le Calvez T, Arzur D, Vandenkoornhuyse P, Barbier G. Diversity of culturable marine filamentous fungi from deep-sea hydrothermal vents. Environ Microbiol. 2009;11: 1588-1600.
14. Burgaud G, Arzur D, Durand L, Cambon-Bonavita MA, Barbier G. Marine culturable yeasts in deep-sea hydrothermal vents: species richness and association with fauna. FEMS Microbiol Ecol. 2010;73: 121-133.
15. Edgcomb VP, Beaudoin D, Gast R, Biddle JF, Teske A. Marine subsurface eukaryotes: the fungal majority. Environ Microbiol. 2011;13: 172-183.
16. Edgcomb VP, Pachiadaki MG, Mara P, Kormas KA, Leadbetter ER, Bernhard JM. Gene expression profiling of microbial activities and interactions in sediments under haloclines of E. Mediterranean deep hypersaline anoxic basins. ISME J. 2016;10: 2643-2657.
17. Ciobanu MC, Burgaud G, Dufresne A, Breuker A, Rédou V, Maamar SB, et al. Microorganisms persist at record depths in the subseafloor of the Canterbury Basin. ISME J. 2014;8: 2352.
18. Rédou V, Ciobanu MC, Pachiadaki MG, Edgcomb V, Alain K, Barbier G, et al. In-depth analyses of deep subsurface sediments using 454-pyrosequencing reveals a reservoir of buried fungal communities at record-breaking depths. FEMS Microbiol Ecol. 2014;90: 908-921.
19. Rédou V, Navarri M, Meslet-Cladière L, Barbier G, Burgaud G. Species richness and adaptation of marine fungi from deep-subseafloor sediments. Appl Environ Microbiol. 2015;81: 3571-3583.
20. Liu CH, Huang X, Xie TN, Duan N, Xue YR, Zhao TX, et al. Exploration of cultivable fungal communities in deep coal-bearing sediments from ∼1.3 to 2.5 km below the ocean floor. Environ Microbiol. 2017;19: 803-818.
21. Burgaud G, Edgcomb VP. (2019)
22. Le Calvez T, Burgaud G, Mahe S, Barbier G, Vandenkoornhuyse P. Fungal diversity in deep sea hydrothermal ecosystems. Appl Environ Microbiol. 2009;75: 6415-6421.
23. Nagahama T, Takahashi E, Nagano Y, Abdel-Wahab MA, Miyazaki M. Molecular evidence that deep-branching fungi are major fungal components in deep-sea methane cold-seep sediments. Environ Microbiol. 2011;13: 2359-2370.
24. Burgaud G, Arzur D, Sampaio JP, Barbier G. *Candida oceani* sp. nov., a novel yeast isolated from a Mid-Atlantic Ridge hydrothermal vent (-2300 meters). Anton van Leeuw. 2011;100: 75-82.
25. Burgaud G, Coton M, Jacques N, Debaets S, Maciel NO, Rosa CA, et al. *Yamadazyma* *barbieri* f.a. sp. nov., an ascomycetous anamorphic yeast isolated from a Mid-Atlantic Ridge hydrothermal site (-2300 m) and marine coastal waters. Int J Syst Evol Microbiol. 2016;66: 3600-3606.
26. Chen CTA, Wang BJ, Huang JF, Lou JY, Kuo FW, Tu YY, et al. Investigation into extremely acidic hydrothermal fluids off Kueishantao islet, Taiwan. Acta Ocean Sin. 2005;24: 125-133.
27. Yang TF, Lan TF, Lee HF, Fu CC, Chuang PC, Lo CH, et al. Gas compositions and helium isotopic ratios of fluid samples around Kueishantao, NE offshore Taiwan and its tectonic implications. Geochem J. 2005;39: 469-480.
28. von Corsel R. A new bathymodioline mussel (Bivalvia: Mytiloidea: Mytilidae: Bathymodiolinae) from vent sites near Kueishan Island, north east Taiwan. Raff Bull Zool. 2008;19: 105-114.
29. Kâ S, Hwang JS. Mesozooplankton distribution and composition on the northeastern coast of Taiwan during autumn: effects of the Kuroshio Current and hydrothermal vents. Zool Stud. 2011;50: 155-163.
30. Wang L, Cheung MK, Liu R, Wong CK, Kwan HS, Hwang JS. Diversity of total bacterial communities and chemoautotrophic populations in sulfur-rich sediments of shallow-water hydrothermal vents off Kueishan Island, Taiwan. Microb Ecol. 2017;73: 571-582.
31. Jiang W, Zhong Y, Shen L, Wu X, Ye Y, Chen CTA, et al. Stress-driven discovery of natural products from extreme marine environment-Kueishantao hydrothermal vent, a case study of metal switch valve. Curr Org Chem. 2014;18: 925-934.
32. White TJ, Bruns T, Lee S, Taylor J. Amplification and direct sequencing of fungal ribosomal RNA genes for phylogenetics. In: Innis MA, Gelfand DH, Sninsky JJ, White TJ, editors. PCR protocols: a guide to methods and application. San Diego: Academic Press; 1990. pp. 315-322.
33. Kumar S, Stecher G, Li M, Knyaz C, Tamura K. MEGA X: molecular evolutionary genetics analysis across computing platforms. Mol Biol Evol. 2018;35: 1547-1549.
34. Martin KJ, Rygiewicz PT. Fungal-specific PCR primers developed for analysis of the ITS region of environmental DNA extracts. BMC Microbiol. 2005;5: 28.
35. Toju H, Tanabe AS, Yamamoto S, Sato H. High-coverage ITS primers for the DNA-based identification of ascomycetes and basidiomycetes in environmental samples. PLoS One 2012;7: e40863.
36. Caporaso JG, Kuczynski J, Stombaugh J, Bittinger K, Bushman FD, Costello EK, et al. QIIME allows analysis of high-throughput community sequencing data. Nat Meth. 2010;7: 335–336.
37. Edgar RC. Search and clustering orders of magnitude faster than BLAST. Bioinformatics 2010;26: 2460-2461.
38. Edgar RC, Haas BJ, Clemente JC, Quince C, Knight R. UCHIME improves sensitivity and speed of chimera detection. Bioinformatics 2011;27: 2194-2200.
39. Kõljalg U, Nilsson RH, Abarenkov K, Tedersoo L, Taylor AFS, Bahram M, et al. Towards a unified paradigm for sequence-based identification of Fungi. Mol Ecol. 2013;22: 5271-5277.
40. Hsieh TC, Ma KH, Chao A. iNEXT: an R package for rarefaction and extrapolation of species diversity (Hill numbers). Meth Ecol Evol. 2016;7: 1451-1456.
41. R Core Team. R: A language and environment for statistical computing. Vienna: R Foundation for Statistical Computing; 2013. Available from: http://www.R-project.org/.
42. Mohamed DJ, Martiny JB. Patterns of fungal diversity and composition along a salinity gradient. ISME J. 2011;5: 379-388.
43. Burgaud G, Woehlke S, Rédou V, Orsi W, Beaudoin D, Barbier G, et al. Deciphering the presence and activity of fungal communities in marine sediments using a model estuarine system. Aquat. Microb. Ecol. 2013;70: 45-62.
44. Pang KL, Jones EBG. Recent advances in marine mycology. Bot Mar. 2017;60: 361-362.
45. Jones EBG, Suetrong S, Bahkali AH, Abdel-Wahab MA, Boekhout T, Pang KL. Classification of marine Ascomycota, Basidiomycota, Blastocladiomycota and Chytridiomycota. Fungal Divers. 2015;3: 1-72.
46. Gunde-Cimerman N, Plemenitas A. Ecology and molecular adaptations of the halophilic black yeast *Hortaea werneckii*. Rev Environ Sci Biotechnol. 2006;5: 323-331.
47. Cabañes FJ, Bragulat MR, Castellá G. *Hortaea werneckii* isolated from silicone scuba diving equipment in Spain. Med Mycol. 2012;50: 852-857.
48. Marchetta A, van den Ende BG, Al-Hatmi AMS, Hagen F, Zalar P, Sudhadham M, et al. Global molecular diversity of the halotolerant fungus *Hortaea werneckii*. Life 2018;8: 31. doi:10.3390/life8030031
49. Sakayaroj J, Preedanon S, Phongpaichit S, Buatong J, Chaowalit P, Rukachaisirikul V. Diversity of endophytic and marine-derived fungi associated with marine plants and animals. In: Jones EBG, Pang KL, editors. Marine fungi and fungal-like organisms. Berlin: De Gruyter; 2012. pp. 291-328.
50. Errasti AD, Novas MV, Carmarán CC. Plant-fungal association in trees: Insights into changes in ecological strategies of *Peroneutypa scoparia* (Diatrypaceae). Flora 2014;209: 704-710.
51. Fell JW, Boekhout T, Fonseca A, Scorzetti G, Statzell-Tallman A. Biodiversity and systematics of basidiomycetous yeasts as determined by large-subunit rDNA D1/D2 domain sequence analysis. Int J Syst Evol Microbiol. 2000;50: 1351-1371.
52. Comeau AM, Vincent WF, Bernier L, Lovejoy C. Novel chytrid lineages dominate fungal sequences in diverse marine and freshwater habitats. Sci Rep. 2016;6: 30120.
53. Xu W, Guo S, Pang KL, Luo ZH. Fungi associated with chimney and sulfide samples from a South Mid-Atlantic Ridge hydrothermal site: distribution, diversity and abundance. Deep-Sea Res. I 2017;123: 48-55.
54. Jones EBG, Sakayaroj J, Suetrong S, Somrithipol S, Pang KL. Classification of marine Ascomycota, anamorphic taxa and Basidiomycota. Fungal Divers. 2009;35: 1-187.
55. Suetrong S, Schoch CL, Spatafora JW, Kohlmeyer J, Volkmann-Kohlmeyer B, Sakayaroj J, et al. Molecular systematics of the marine Dothideomycetes. Stud Mycol. 2010;64: 155-173.
56. Jones EBG, Choeyklin R. Ecology of marine and freshwater basidiomycetes. Brit Mycol Society Symp Ser. 2008;28: 301-324.
57. Spiers AG, Brewster DT. Evaluation of chemical and biological treatments for control of *Chondrostereum purpureum* infection of pruning wounds in willows, apples, and peaches. New Zeal J Crop Hort Sci. 1997;25: 19-31.
58. Fröhlich-Nowoisky J, Burrows SM, Xie Z, Engling G, Solomon PA, Fraser MP, et al. Biogeography in the air: fungal diversity over land and oceans. Biogeosciences 2012;9: 1125-1136.
59. Cannon PF, Damm U, Johnston PR, Weir BS. (2012) *Colletotrichum*–current status and future directions. Stud Mycol. 2012;73: 181–213.
60. Reeb D, Best PB, Botha A, Cloete KJ, Thornton M, Mouton M. Fungi associated with the skin of a southern right whale (*Eubalaena australis*) from South Africa. Mycology 2010;1: 155-162.
61. Jebaraj CS, Raghukumar C, Behnke A, Stoeck T. Fungal diversity in oxygen-depleted regions of the Arabian Sea revealed by targeted environmental sequencing combined with cultivation. FEMS Microbiol Ecol. 2010;71: 399-412.
62. Bonifaz A, Badali H, de Hoog GS, Cruz M, Araiza J, Cruz MA, et al. Tinea nigra by *Hortaea werneckii*, a report of 22 cases from Mexico. Stud Mycol. 2008;61: 77-82.
63. Sue PK, Gurda GT, Lee R, Watkins T, Green R, Memon W, et al. First report of *Westerdykella dispersa* as a cause of an angioinvasive fungal infection in a neutropenic host. J Clin Microbiol. 2014;52: 4407-4411.
64. Tsang CC, Chan JFW, Pong WM, Chen JHK, Ngan AHY, Cheung M, et al. Cutaneous hyalohyphomycosis due to *Parengyodontium album* gen. *et* comb. nov. Med Mycol. 2016;54: 699-713.
