# Supplementary material for: Insights into fungal diversity of a shallow-water hydrothermal vent field at Kueishan Island, Taiwan by culture-based and metabarcoding analyses
Source: PLoS One. 2019 Dec 30;14(12):e0226616. doi: 10.1371/journal.pone.0226616 (PMC6936883; doi:10.1371/journal.pone.0226616)
Supplement: Attachment — Submitted filename: Response to Reviewers.docx [file pone.0226616.s004.docx]

4 October 2019
Clement Kin-Ming Tsui

Associate Editor

PLoS One

Dear Dr. Tsui,

We are submitting a revised manuscript entitled ‘Insights into fungal diversity of a shallow-water hydrothermal vent field at Kueishan Island, Taiwan by culture-based and metabarcoding analysis’. We have attended to all comments made by you and the reviewer and made corresponding changes to the manuscript. We have also reformat the manuscript based on the journal requirements. Here we list the response to the comments:

Associate Editor

Comment 1: ‘*line 215-216 - although the amplicons were sent to a commercial platform, it was essential to describe the library preparation method and the package for sequencing (e.g. Miseq V2 or V3). How many cycles of sequencing reaction?*’

Response: TruSeq DNA Nano (input 120 ng / PCR 5 cycles) was used for library preparation. Miseq v3 was used and 600 cycles was used in the sequencing reaction.

Comment 2: ‘*line 231-242 - the description of statistical analysis was inadequate and incomplete. Use R packages or other statistical software instead of MS Excel; Different analysis packages/tools should be used for culture-based and sequence based data. Have you estimated the alpha, beta diversity (similar to Bray-Curtis dissimilarity) for the amplicon data?*’

Response: Figure 6 has been replaced by a new figure showing a heatmap of the Bray-Curtis distances between the five sediment samples (beta diversity).

Comment 3: ‘*line 295-296 - report the read and sequence information: read length before and after quality trimming; # of chimera reads; # of reads due to contamination (there was a negative control)*’

Response: The average read lengths before quality trimming were 1 to 273 base pair (bp) and reads of 271-273 bp used for the analyses. Control PCRs (water control) were performed but no PCR products were obtained and therefore, these samples were not sequenced for comparison. These information is added to the new Supplementary Table 2 and the text.

Comment 4: ‘*line 296 - there was 10 fold difference in # of reads among samples; have you normalized the # of reads before the data analysis. Did you observe any changes in the communities with or without normalization of reads?*’

Response: We agree with the editor that normalization is required for the data due to the fold differences between samples. We have re-run the PCR analysis with percentage normalization and Figure 4 has been replaced.

Comment 5: ‘*Fig.4, line 440-444 - sample B15-b was an outlier. What could be the possible ecological factors or technical reasons? Another PCA diagram should be generated without B15-b to show the variation among other samples (The current Fig 4 was not acceptable). Otherwise the variations (temporal or substrate specificity) could be masked.*’

Response: The PCA was re-run with normalization of reads including the sample B15-b. B15-b clusters with other samples. The outlier is B16-b but only in PC1 which only constitutes 16.8% of the variation. Figure 4 has been replaced.

Comment 6: ‘*Fig.6 - the samples were collected from 3 locations; How much observed variation among the communities was due to geographic factor? Mantel test or a correlation between community variation and geographic distance should be established.*’

Response: A Mantel test was run and was found that there was a low correlation among the spatial distance and the ecological distance with negatively association (r = -0.1098). Species composition and sampling sites were concluded to be unrelated (p = 0.55). These information has been added to the text.

Comment 7: ‘*line 128, 438 - if the primers were biased towards the higher fungi and if the study did not aim at lower fungi, this should be mentioned in the introduction/ materials and methods.*’

Response: This has been added in the Materials and Methods and it reads ‘These primers were found to amplify sequences of the Ascomycota and the Basidiomycota.’

Comment 8: ‘*line 198-199 - the ITS sequences are deposited; however the raw reads also need to be deposited in the GenBank / SRA.*’

Response: The ITS sequences from the isolation study and the raw reads from the metabarcoding study have been deposited in GenBank/SRA and their accession numbers have been added to the manuscript.

Reviewer 1
Comment 9: ‘*The fungal diversity reported from this shallow hydrothermal vent system is valuable, but the impact of the paper can be significantly elevated by simply incorporating relevant environmental parameters such as temperature, depth, oxygen concentration, sulphide concentration, sediment porosity, water turbidity, primary productivity, etc. Are any of these variables measured at the site at the time of sample collection? If not, is there any historic data reported in the literature that can be used for interpreting the fungal diversity? One major parameter that was repeatedly referred to in the manuscript is the sediment type (yellow vs. black). What caused the sediment to have different colors? How do the physical and chemical properties of the two types of sediments compare? Do they have the same porosity? What is the nutrient concentration in the sediment porewater? What is the depth of each sediment sampled? Similarly, what are the differences between the three sampling sites? Is the yellow sediment from location V the same as the yellow sediment in location E? The multiple locations of sampling are a strength of this study, and it will be of high interest for microbial ecologists to learn which factor played the most important role in determining the fungal community. Was it the sediment type or the physicalchemical variables associated with each location? Specifically, the authors can perform statistical tests to address this question.*’

Response: The characteristics of the sediment samples have been added in the text and it reads ‘Visible sulfur granules were found in the yellow sediment samples (with a higher SO_3_ content) but not in the black sediment samples (with a lower SO_3_ content).’ The depth of the sampling sites was also provided in Table 1. However, the other information regarding environmental variable and sample properties mentioned by the reviewer is not available from us or literature.

Comment 10: ‘*Moreover, the discussion of the manuscript is inadequate. Since other marine systems have been mentioned in the introduction, it is natural and necessary to compare the fungal diversity discovered at the shallow hydrothermal vent to other systems such as deep hydrothermal vents. At which location is fungal diversity higher? How important is depth and organic matter input in determining the diversity of marine fungi?*’

Response: A discussion between the results of this study and others from the deep-sea has been added. Most studies of fungal diversity of hydrothermal vent ecosystem are from the deep-sea and this, to our knowledge, is the first study of fungal diversity of a shallow hydrothermal vent ecosystem. More information on the latter is needed to make a comparison between the two types of hydrothermal vents. Currently, little information is available to relate depth and organic matter input on the diversity of marine fungi.

Comment 11: ‘*The introduction in its current form requires major revision. The lengthy second paragraph of the introduction is a brief review of deep-sea fungi including those from deep-sea hydrothermal vents, which has little relevance to the current study. I suggest removing the majority of the contents from this paragraph and condense it to a summary of marine habitats where marine fungal diversity has been surveyed. At the end it should be pointed out that fungal diversity in shallow hydrothermal vent system is a gap in our knowledge.*’

Response: The introduction has been reorganized as suggested by the reviewer. It now only summarizes brief results of fungal diversity from other deep-sea hydrothermal vent studies.

Comment 12: ‘*Additionally, the method and/or the result section needs to include a few key pieces of information. As for the metabarcoding analysis of sediments, how many reads were merged? How many reads can be classified as fungal? How many reads were classified as other known taxa? How many reads cannot be classified as any known taxa? What is the classification threshold? As for PCA, how were data transformed before ordination? What is the definition of species composition?*’

Response: Species composition includes both species richness and abundance of each species. For the PCA, the reads were transformed into % abundance (reads of each species over total reads × 100%) for each species. The number of removed reads, merged reads, fungal reads and reads classified as other known taxa are provided in Supplementary Table 2. These information has been added to the text.

Comment 13: ‘*The discussion section should include comparison with other studies on deep-sea hydrothermal vents as mentioned above, and a discussion on what environmental variables may have played a role in determining the fungal diversity. The current section “Putative ecological roles” is a misnomer because the majority of this section deals with either diversity alone or dispersal mechanisms. The section on Malassezia is really about its biogeography and not about its ecological roles. Therefore, I suggest restructuring the section into biogeography and dispersal mechanisms.*’

Response: The section title has been changed from ‘Putative ecological roles’ to ‘Ecology of fungi’. A discussion between the results of this study and others from the deep-sea has been added. Jones (2000) has reviewed the key factors (temperature, salinity, etc.) influencing diversity of marine fungi but concerned only those occurring on organic substrates (mainly wood) in coastal habitats.

Comment 14: ‘*“taxa” (line 33) is a vague term and should be avoided when possible. In the manuscript, taxa refer to species/genus in most occasions, and should be revised accordingly.*’

Response: The term ‘taxa’ has been changed to ‘species’ in most cases, but is kept where necessary.

Comment 15: ‘*Line 64-65: it is misleading to state “especially in the deep sea”. I suggest removing this part.*’

Response: This sentence has been modified and it reads ‘Yet, marine fungi are not represented in ocean ecosystem models [7], despite growing evidence of diverse marine fungi in the ocean.’

Comment 16: ‘*Line 135: (The section on Collection of Samples). Table 1 should include information about which site and which type of substrate was sampled for cultivation.*’

Response: These information is now available in Table 1.

Comment 17: ‘*Line 220: “paired” should be “merged”*.’

Response: This has been corrected.

Comment 18: ‘*Line 256: “taxa” should be “species”.*’

Response: This and others have been corrected.

Comment 19: ‘*Table 2: what does the asterisk sign mean? It needs to be explained in the caption.*’

Response: This is explained in the caption.

Comment 20: ‘*Figure 3: it needs to be specified which sub-figures are for cultivation results and which sub-figures are for metabarcoding results.*’

Response: These have already been specified in the y-axis and explained in the caption. No change is required.

Reviewer 2

Comment 21: ‘*Please don't use the term culturomics! I would use culture-based through out the manuscript instead.*’

Response: This term has been changed throughout.

Comment 22: ‘*Additional coorections are annotated on the attached manuscript.*’

Response: These have been corrected.

Comment 23: ‘*Please include a summary or conclusion section at the end if possible.*’

Response: A conclusion section is added and it reads ‘Fungal diversity in shallow hydrothermal vent system is a gap of our knowledge of the marine mycota. In this study, 54 and 49 species of the Ascomycota and the Basidiomycota were found in the black and yellow sediments collected at/near the shallow hydrothermal vent area of Kueishan Island by culture-based and metabarcoding methods, suggesting a high diversity of fungi in this extreme environment.’
