# Supplementary material for: Insights into fungal diversity of a shallow-water hydrothermal vent field at Kueishan Island, Taiwan by culture-based and metabarcoding analyses
Source: PLoS One. 2019 Dec 30;14(12):e0226616. doi: 10.1371/journal.pone.0226616 (PMC6936883; doi:10.1371/journal.pone.0226616)
Supplement: Attachment — Submitted filename: PONE-D-19-20402 review.docx [file pone.0226616.s005.docx]

PONE-D19-2040 review

This is an interesting contribution to marine mycology and ecology and I would recommend publication. However, there is a methodological issue which needs to be highlighted in the paper. Ideally the crab and egg samples would have been surface-sterilised before being ground up for culturing and molecular analysis. The results are still interesting and worth reporting, but it is important to discuss the fact that the cultures and DNA from these samples may simply be from spores resting on the surface of the crabs.

The authors make a very valid point in the discussion about airborne spores being found when using the metabarcoding methods. This is a really important point which is often missed in metabarcoding studies. It would be good to mention this point in the abstract.

Introduction:

First sentence is too long. Please break it up.

Please define "deep sea".

Please define "deep subsurface". How far below surface?

Lines 59 to 60: Change " fungal species appears associated to the marine environment with 1255 species recently documented from the ocean [5]." to "fungal species appear to be associated with the marine environment with just 1255 species...".

Line 71: Change "... depending on the habitat, are thus facing many..." to "... depending on the habitat, thus face many...

Line 72: Change "...gradients, variable sea salt..." to "gradients and variable sea salt..."

Line 97: The word "illustrated" here doesn't seem right. Perhaps just use "found".

Line 105: Change "...vents appear as unique..." to "...vents appear to be unique..."

Line 115: Change "28-29], however, knowledge..." to "28-29]. However, knowledge...".

Materials and methods:

Ideally the crabs and eggs would have been surface sterilised before culturing and molecular analysis.

Line 143: Were the universal bottles sterilised?

Line 146-147: How were the samples divided? Was there any randomisation process? If so, then please add this.

Table 1: Ideally all samples that are to be compared would be collected on the same day.

Line 174: Why was 25 deg C chosen? How does this compare to the temperature where the samples were taken?

Line 175: Change "appeared" to "appearing".

Line 184: Change "scrapped" to "scraped".

Line 203: Change "Maxi Kit" to "Maxi Kits".

Results:

Line 285: Change "were" to "was"

Line 291: Change "subjected to the DNA..." to "subjected to DNA..."

Line 297: Change "operational taxonomic unit" to "operational taxonomic units".

Line 314: Change "based the" to "based on the".

Discussions:

Line 395: Change " associated to" to "associated with".

Line 440: Change "Fungal community" to "Fungal communities".

Line 443: Change "habor" to "habors".

Line 463: Change "majority has been" to "majority have been".

Line 484 to 486: This is an interesting point. Please expand on this and provide references for the reader to follow up.
