# Supplementary material for: Insights into fungal diversity of a shallow-water hydrothermal vent field at Kueishan Island, Taiwan by culture-based and metabarcoding analyses
Source: PLoS One. 2019 Dec 30;14(12):e0226616. doi: 10.1371/journal.pone.0226616 (PMC6936883; doi:10.1371/journal.pone.0226616)
Supplement: Attachment — Submitted filename: Response to Reviewers 26 Nov 2019.docx [file pone.0226616.s006.docx]

Editor’s Comments:

**Comment**: ‘*What would happen when sample 16-b (in PCA1) was excluded from the analysis? Have you done the comparison?*’

**Response**: We have actually done the analysis without B16-b (as shown below) and both axes were found to contribute significant variations in the sample B16-a. These suggest the samples collected at Site E were with very different species composition. We have modified the text as ‘PC1 axis contributed significantly to one of the two black sediment samples at site E (B16-b). If the outliner B16-b was removed from the analysis, both PC1 and PC2 contributed significantly to the other black sediment sample at site E (B16-a) (results not shown).’

Reviewer 1:

**Comment 1**: ‘*However, there is a methodological issue which needs to be highlighted in the paper. Ideally the crab and egg samples would have been surface-sterilised before being ground up for culturing and molecular analysis. The results are still interesting and worth reporting, but it is important to discuss the fact that the cultures and DNA from these samples may simply be from spores resting on the surface of the crabs.*’

**Response**: We agree with the reviewer. A sentence has been added in the discussion and it reads ‘It is also possible that the cultures isolated from the crabs (and the animal eggs) represented resting spores on the crab surface at the time of collection.’

**Comment 2**: ‘*The authors make a very valid point in the discussion about airborne spores being found when using the metabarcoding methods. This is a really important point which is often missed in metabarcoding studies. It would be good to mention this point in the abstract.*’

**Response**: A sentence has been added in the abstract and it reads ‘While some fungi found in this study were terrestrial species and their airborne spores might have been deposited into the marine sediment,…………’

**Comment 3**: ‘*Introduction First sentence is too long. Please break it up.*’

**Response**: The first sentence has been broken up into two sentences.

**Comment 4**: ‘*Please define "deep sea".*’

**Response**: The ‘deep-sea’ was loosely defined as ‘habitats below the epipelagic zone, (Herring 2001) and this has been added to the text.

**Comment 5**: ‘*Please define "deep subsurface". How far below surface?*’

**Response**: The deep subsurface habitats include both sedimentary and oceanic crustal habitats and this has been added to the text.

**Comment 6**: ‘*Lines 59 to 60: Change " fungal species appears associated to the marine environment with 1255 species recently documented from the ocean [5]." to "fungal species appear to be associated with the marine environment with just 1255 species...".*’

**Response**: This has been changed.

**Comment 7**: ‘*Line 71: Change "... depending on the habitat, are thus facing many..." to "... depending on the habitat, thus face many...*’

**Response**: This has been changed.

**Comment 8**: ‘*Line 72: Change "...gradients, variable sea salt..." to "gradients and variable sea salt..."*’

**Response**: This has been changed.

**Comment 9**: ‘*Line 97: The word "illustrated" here doesn't seem right. Perhaps just use "found".*’

**Response**: I cannot find the word ‘illustrated’ in the manuscript in Line 97.

**Comment 10**: ‘*Line 105: Change "...vents appear as unique..." to "...vents appear to be unique..."*’

**Response**: This has been changed.

**Comment 11**: ‘*Line 115: Change "28-29], however, knowledge..." to "28-29]. However, knowledge...".*’

**Response**: This has been changed.

**Comment 12**: ‘*Materials and methods: Ideally the crabs and eggs would have been surface sterilised before culturing and molecular analysis.*’

**Response**: See Comment 1.

**Comment 13**: ‘*Line 143: Were the universal bottles sterilised?*’

**Response**: Yes, the universal bottles were sterile. The word ‘sterile’ is added before universal bottles in the text.

**Comment 14**: ‘*Line 146-147: How were the samples divided? Was there any randomisation process? If so, then please add this.*’

**Response**: No randomisation process was performed. The text has been modified to clarify how the samples were divided and it reads ‘For each of the twenty-two sediment subsamples (in universal bottles) collected on the five collection dates, half of the sediment was transferred aseptically to another sterile universal bottle and freeze-dried for DNA extraction while the other half was used for isolation.’

**Comment 15**: ‘*Table 1: Ideally all samples that are to be compared would be collected on the same day.*’

**Response**: We agree with the reviewer on this. In fact, we have analysed twenty-two sediment subsamples in the metabarcoding analysis, but positive results were only obtained from 13 subsamples collected on the five collection dates. This is mentioned in the discussion.

**Comment 16**: ‘*Line 174: Why was 25 deg C chosen? How does this compare to the temperature where the samples were taken?*’

**Response**: From January 2014 to December 2017, the average water temperatures at Kueishan Island were between 20.2 °C and 29.4 °C. The use of 25 °C to incubate the inoculated samples was based on roughly the median of the lowest and highest average water temperatures.

**Comment 17**: ‘*Line 175: Change "appeared" to "appearing".*’

**Response**: This has been changed.

**Comment 18**: ‘*Line 184: Change "scrapped" to "scraped".*’

**Response**: ‘Scraped’ was used in the original text.

**Comment 19**: ‘*Line 203: Change "Maxi Kit" to "Maxi Kits".*’

**Response**: This has been changed.

**Comment 20**: ‘*Results: Line 285: Change "were" to "was"*’

**Response**: This has been changed.

**Comment 21**: ‘*Line 291: Change "subjected to the DNA..." to "subjected to DNA..."*’

**Response**: This has been changed.

**Comment 22**: ‘*Line 297: Change "operational taxonomic unit" to "operational taxonomic units".*’

**Response**: This has been changed.

**Comment 23**: ‘*Line 314: Change "based the" to "based on the".*’

**Response**: ‘based on the’ was used in the original text.

**Comment 24**: ‘*Discussions: Line 395: Change " associated to" to "associated with".*’

**Response**: ‘associated with’ was used in the original text.

**Comment 25**: ‘*Line 440: Change "Fungal community" to "Fungal communities".*’

**Response**: This has been changed.

**Comment 26**: ‘*Line 443: Change "habor" to "habors".*’

**Response**: This has been changed.

**Comment 27**: ‘*Line 463: Change "majority has been" to "majority have been".*’

**Response**: This has been changed.

**Comment 28**: ‘*Line 484 to 486: This is an interesting point. Please expand on this and provide references for the reader to follow up.*’

**Response**: We have elaborated on the topic and the text reads as ‘Occurrence of filamentous basidiomycetes in this report and in other studies may suggest that spores of the terrestrial Polyporales (e.g. *Bjerkandera adusta*, *Cerrena* sp., *Phanerochaete tuberculate*, *Phlebia chrysocreas*, *Rigidoporus* sp., *Trametes cubensis* and *T. versicolor*) and Agaricales (e.g. *Chondrostereum* sp. and *Schizophyllum commune*) deposit in the marine sediment [19−20]. Kueishan Island is only ~10 km away from the main Taiwan Island and it is likely that spores of the filamentous basidiomycetes were originated from the terrestrial environment through freshwater runoff or air deposition [61] and deposited into the sediment.’

Reviewer 2:

**Comment 29**: ‘*In the abstract: 1. The metabarcoding analysis amplifying a small fragment of the rDNA (from 18S to 5.8S) recovered 7-27 species from the black sediment and 12-27 species from the yellow sediment samples of the Ascomycota and the Basidiomycota. Add respectively at the end with a comma.*’

**Response**: The meaning of the sentence is not clear as written. This has been rewritten as ‘The metabarcoding analysis amplifying a small fragment of the rDNA (from 18S to 5.8S) recovered 7-27 species from the black sediment and 12-27 species from the yellow sediment samples and all species belonged to the Ascomycota and the Basidiomycota.’

**Comment 30**: ‘*2. In the abstract: One or two sentences to put results in a general context and broader perspective are missing.*’

**Response**: An extra sentence has been added and it reads ‘This study is the first to observe a high diversity of fungi associated various substrates at a marine shallow water hydrothermal vent ecosystem.’

**Comment 31**: ‘*3. Line 284 One common species were should be "one common species was isolated.*’

**Response**: This has been changed.

**Comment 32**: ‘*4. Figure legends are still not explaining the key results. Doing so will make the paper more interesting to the reader.*’

**Response**: The figure legends have been elaborated.

**Comment 33**: ‘5. Conclusion statement is weak, please strengthen it.’

**Response**: The conclusion has been rewritten.
